# Higher Prevalence of PldA, a *Pseudomonas aeruginosa Trans*-Kingdom H2-Type VI Secretion System Effector, in Clinical Isolates Responsible for Acute Infections and in Multidrug Resistant Strains

**DOI:** 10.3389/fmicb.2018.02578

**Published:** 2018-10-29

**Authors:** Thibaud Boulant, Yves-Marie Boudehen, Alain Filloux, Patrick Plesiat, Thierry Naas, Laurent Dortet

**Affiliations:** ^1^EA7361 Structure, Dynamic, Function and Expression of Broad Spectrum β-Lactamases, LabEx Lermit, Faculty of Medicine, Université Paris Sud – Université Paris Saclay, Le Kremlin-Bicêtre, France; ^2^MRC Centre for Molecular Microbiology and Infection, Department of Life Sciences, Imperial College London, London, United Kingdom; ^3^Bacteriology Unit, University Hospital of Besançon, Besançon, France; ^4^Bacteriology-Hygiene Unit, Assistance Publique/Hôpitaux de Paris, Bicêtre Hospital, Le Kremlin-Bicêtre, France; ^5^Associated French National Reference Center for Antibiotic Resistance: Carbapenemase-producing Enterobacteriaceae, Le Kremlin-Bicêtre, France

**Keywords:** type III secretion system, type VI secretion system, PldA, PldB, Exo toxins, multidrug resistance, carbapenemase, IMP

## Abstract

*Pseudomonas aeruginosa* can manipulate eukaryotic host cells using secreted effectors delivered by the type III or the type VI Secretion Systems (T3SS and T6SS). The T3SS allows the injection of bacterial effectors (Exo toxins) into eukaryotic cell. *P. aeruginosa*, encodes three T6SSs, H1-, H2- and H3-T6SS. The H1-T6SS is mainly involved in delivering toxins to kill bacterial competitors. Recently, two T6SS-secreted phospholipases D, PldA (H2-T6SS) and PldB (H3-T6SS), were identified as *trans*-kingdom virulence effectors, triggering both killing of bacterial competitors and internalization into non-phagocytic cells. We deciphered the prevalence of T3SS and T6SS effectors encoding genes in 185 clinical isolates responsible for infections (septicaemia, pulmonary infections, urinary tract infections, and chronic infections in CF patients), 47 environmental strains, and on 33 carbapenemase-producers. We included 107 complete genomes of *P. aeruginosa* available in public databases. The prevalence of *pldA* is increased in clinical isolates responsible for severe acute infection and particularly in multi-drug resistant strains. In contrast, the *pldB* prevalence was high (96.8%) in all isolates. Regarding T3SS effectors, *exoT* and *exoY* are present in nearly all isolates while *exoS* and *exoU* were found to be exclusive with a higher prevalence of *exoU*^+^ strains in severe acute infections. The hypervirulent *exoU*^+^ isolates are more prone to be *pldA*^+^, suggesting a role of PldA in virulence. Finally, we observed that extremely drug resistant isolates producing an IMP-type carbapenemase were all *pldA*^+^. Our results suggest that PldA might have a role during pulmonary infections and have been co-selected in multidrug resistant strains particularly IMP-producers.

## Introduction

*Pseudomonas aeruginosa* is an extracellular Gram-negative bacterium responsible for more than 51,000 healthcare associated infections per year in the United States according to Centers for Disease Control and Prevention (2013). *P. aeruginosa* related-infections are mostly healthcare associated and cause a large subset of diseases including urinary tract infections (UTIs), acute pulmonary infections, wound infections, acute otitis and septicaemia (Stryjewski and Sexton, 2003). It is also a major cause of chronic infection in patients with cystic fibrosis (CF) (Lund-Palau et al., 2016). Indeed, this opportunistic pathogen is responsible of 85% chronic infections in the CF patient during adolescence and its propagation is involved in the progression of lung damage in CF patients (Davies, 2002).

Like many other Gram-negative bacteria, *P. aeruginosa* is able to manipulate eukaryotic host cells using its type III secretion system (T3SS) (Dortet et al., 2018) and more precisely secreted exoenzymes ExoS, ExoT, ExoY, and ExoU (Cornelis, 2006; Galle et al., 2012). More recently the type VI secretion system (T6SS) was shown to deliver bacterial toxins. The genome of *P. aeruginosa* encodes three T6SSs (H1-, H2-, and H3-T6SS) (Hachani et al., 2016). The H1-T6SS and its main effectors, Tse1-3, has been demonstrated to have a crucial role in bacterial warfare and thus possibly help to outcompete and manipulate the resident microbiota in the host (Cianfanelli et al., 2016). At the opposite, very little is known about H2- and H3-T6SS. Recently, a novel class of T6SS effectors that trigger both killing of bacterial competitors and internalization into non-phagocytic cells have been identified in *P. aeruginosa* (Russell et al., 2013). The T6SS-secreted phospholipases D (Pld), PldA and PldB, are thus the first example of *trans*-kingdom virulence effectors (Russell et al., 2013; Jiang et al., 2014). PldA and PldB have been proposed to be secreted via H2- and H3-T6SS, respectively (Russell et al., 2013; Jiang et al., 2014; Hachani et al., 2016). These two virulence factors are encoded in two distinct *pld* clusters that are not present in all *P. aeruginosa* isolates (Wilderman et al., 2001).

*Pseudomonas aeruginosa* is on the World Health Organization list of antibiotic resistant priority pathogens. Indeed, *P. aeruginosa* is intrinsically resistant to a number of antimicrobials due to the low permeability of its outer-membrane, the constitutive expression of various efflux pumps, and the production of β-lactamases (El Zowalaty et al., 2015). During the last decade, multidrug-resistant (MDR) isolates have been increasingly reported worldwide. The rise of multidrug resistance in *P. aeruginosa* is linked to the dissemination of extended-spectrum β-lactamases (ESBLs) and more recently of carbapenemase-producing isolates (Mesaros et al., 2007). Currently, the most widespread carbapenemases in *Pseudomonas* spp. are metallo-β-lactamases of VIM- (Verona imipenemase) and IMP- (Imipenemase) types (Gupta, 2008; Miriagou et al., 2010). Of note, VIM- and IMP-producers are usually resistant to all antimicrobial molecules except polymyxins and sometimes aztreonam, resulting in critical therapeutic issues in case of severe infections, such as septicaemia and pneumonia (Barbier et al., 2013). Thus, according to the CDC report, infections caused by these MDR *P. aeruginosa* represents 13% (6,700 / 51,000) of *P. aeruginosa* related infections and were responsible to 440 deaths per year in the United States in 2013 (Centers for Disease Control and Prevention, 2013).

In the context of the rapid emergence of pan-drug resistant *P. aeruginosa* isolates, the development of alternative ways to treat infected patients is crucial. Virulence inhibition strategies seem to interesting approaches. A prerequisite is, however, to establish among clinical isolates the prevalence of virulence factors encoding genes. Although the prevalence and the role of the T3SS effectors (ExoS, ExoT, ExoY, and ExoU) has been extensively described in clinical isolates, the prevalence of T6SS effectors, and particularly the *trans*-kingdom phospholipases PldA and PldB, remain less understood. Here, we determined the prevalence of genes encoding T3SS and T6SS effectors, including phospholipase PldA and PldB, in (i) *P. aeruginosa* isolates responsible for human infections (UTIs, acute pulmonary infection, septicaemia, CF-related chronic infections) compared to environmental strains, and (ii) in a collection of MDR *P. aeruginosa* isolates including the most relevant worldwide disseminated MDR clones [e.g., the VIM-2 producing clone of sequence type (ST) 11 and the IMP-13-producing clone of ST 621] (Santella et al., 2010).

## Materials and Methods

### Strain Collection

A collection of 185 *P. aeruginosa* isolates responsible for human infections were collected at the Bicêtre and Besançon hospital from 2016 to 2017. These infections included septicaemia (*n* = 46), UTIs (*n* = 32), pulmonary infections (*n* = 58) and chronic infections in CF patient (*n* = 49). The 58 pulmonary infections were acute infections occurring in patients deprived of any chronic pulmonary underlying diseases (e.g., CF patients, obstructive bronchopneumopathy). In addition, 47 environmental isolates from Besançon area were also tested. These environmental isolates were all recovered from natural setting without any link with hospital effluents. Finally, an additional collection of 33 well-characterized MDR *P. aeruginosa* isolates was also included in this study. These MDR isolates were all carbapenemase-producers including KPC-2 (*n* = 4); two GES-type (GES-2 and GES-5) producers; 10 VIM-producers (VIM-1, -2, and -4), 9 IMP-producers (IMP-1, -2, and -13), one NDM-1 producer; 3 GIM-1 producers; 3 AIM-1 producers and one SPM-1 producer.

### Antimicrobial Susceptibility Testing

Antimicrobial susceptibility was determined by disk diffusion on Mueller Hinton (MH) agar plates (Biorad, Marnes la Coquette, France) at 37°C. All results were interpreted using EUCAST breakpoint as updated in 2017^1^.

### Molecular Detection of T3SS and T6SS Effectors Encoding Genes

PCR assays were performed for detection of the *exoU*, *exoS*, *exoT*, and *exoY* (T3SS effectors encoding genes), *tse1, tse2*, and *tse3* (H1-T6SS effectors encoding genes), *pldA* and *pldB* (H2- and H3-T6SS effectors encoding genes, respectively). PCR amplification of structural component encoding genes of T3SS (*pscC*) and H1-, H2-, H3-T6SS (*tssB1, tssB2*, and *tssB3*, respectively) were performed to confirm the presence or absence of these secretion machineries. DNA extracts were obtained by boiling method. Briefly, one bacterial colony was mixed in 100 μl of sterile water and boiled at 100°C for 10 min. Bacteria residues were pelleted by centrifugation at 13000 *g* for 5 min. The DNA extract was present in the supernatant. All primers used in this study are indicated in Table 1. The PCR mix was as follow: 2 μl of DNA extract obtained by boiling extract, 2.5 μl of reverse and forward primers, 5 μl of 10X PCR buffer, 1 μl of dNTP, 0.4 μl Taq DNA polymerase (5 U/μl; Roche) and 36.6 μl of water. The PCR protocol was as follow : 5 min initial denaturation at 95°C; 35 cycles including 30 s denaturation at 95°C, 1 min hybridization at 55°C and 1 min elongation at 72°C; and a final elongation step at 72°C for 10 min. Amplification PCR products were visualized using an UV transilluminator after electrophoresis in 2% agar gel for 40 min at 80 V.

**Table 1:** List or primers used for the detection of T3SS and T6SS effectors.

Secretion system	Gene	Primers	
		Name	Sequences
T3SS	*pscC*^∗^	*F pscC*	5′-ccaggcctgccttacgactat-3′
		*R pscC*	5′-tcgtagtaccagcccgaggtt-3′
	*exoS*	*R exoS*	5′-atgccggtgtagagaccaag-3′
		*F exoS*	5′-aggtcagcagagtatcggc-3′
	*exoT*	*R exoT*	5′-tcgatcgatatctcgctgac-3′
		*F exoT*	5′-tgaacaggtcgtgaagacc-3′
	*exoU*	*R exoU*	5′-acgtaacagagctacgttgg-3′
		*F exoU*	5′-ttgatcatcagcagttactcg-3′
	*exoY*	*R exoY*	5′-tgccatagaatccgtcctcg-3′
		*F exoY*	5′-atgacctcttcctggtagcg-3′
H1-T6SS	*tssB1*^∗^	*F tssB1*	5′-gtgcagatcgagtacgacg-3′
		*R tssB1*	5′-tcttgccgtccatgtaggt-3′
	*tse1*	*R tse1*	5′-agggagtagacgtagtag-3′
		*F tse1*	5′-aagtacccgatgtgctggtg-3′
	*tse2*	*R tse2*	5′-acgaagttgtccagttgg-3′
		*F tse2*	5′-ttaaggtgaagcaggacagc-3′
	*tse3*	*R tse3*	5′-ttgctgtacttggtgacgg-3′
		*F tse3*	5′-atccagggtccgttcag-3′
H2-T6SS	*tssB2*^∗^	*F tssB2*	5′-atggccaaagaaggctcggt-3′
		*R tssB2*	5′-tggtgcttggccaggacctc-3′
	*pldA*	*R pldA*	5′-tgtcgatcaccacggatttc-3′
		*F pldA*	5′-tatgacttcgaaaccatgctcg-3′
H3-T6SS	*tssB3*^∗^	*F tssB3*	5′-gtacgcagcacaagctggac-3′
		*R tssB3*	5′-ttgaccgggtcgaagtcctc-3′
	*pldB*	*R pldb*	5′-ttcgatgttgttttcctggcc-3′
		*F pldb*	5′-aagaccacctacgacatgatg-3′

^∗^*genes encoded a structural protein of the secretion system (T3SS or T6SS)*.

### *In silico* Detection of T3SS and T6SS Effectors in Complete Genomes of *P. aeruginosa*

From the European Nucleotide Archive (ENA) 107 complete genome of *P. aeruginosa* were identified and analyzed. Among these 107 *P. aeruginosa* strains, 20 were carbapenemase-producers including 2 GES-20, 2 IMP-1, 2 IMP-34, 3 NDM-1, 1 DIM-1, 5 SPM-1, and 5 VIM-2. T3SS/T6SS effectors encoding genes were searched with the Basic local alignment search tool nucleotide (BLASTn) program on *Pseudomonas* genome website^2^ using *P. aeruginosa* PAO1 and UCBPP-PA14 as reference genomes.

### Statistical Data Analysis

Statistical analysis was performed using GraphPad Prism v6 software. Data were compared using χ^2^ test. *P*-values < 0.05 were considered statistically different.

## Results

### Prevalence of Genes Encoding T3SS and T6SS Effectors in *P. aeruginosa*

All the 339 *P. aeruginosa* isolates (185 clinical isolates, 47 environmental isolates, and 107 complete genome of *P. aeruginosa*) were positive for genes encoding structural components of T3SS (*pscC*) and H1-, H2-, and H3-T6SS (*tss1*, *tss2*, *tss3*, respectively) except for two strains recovered from a CF patient, which gave negative results for *tssB3* and one environmental strain that was negative for *pscC* gene.

**FIGURE 1:**
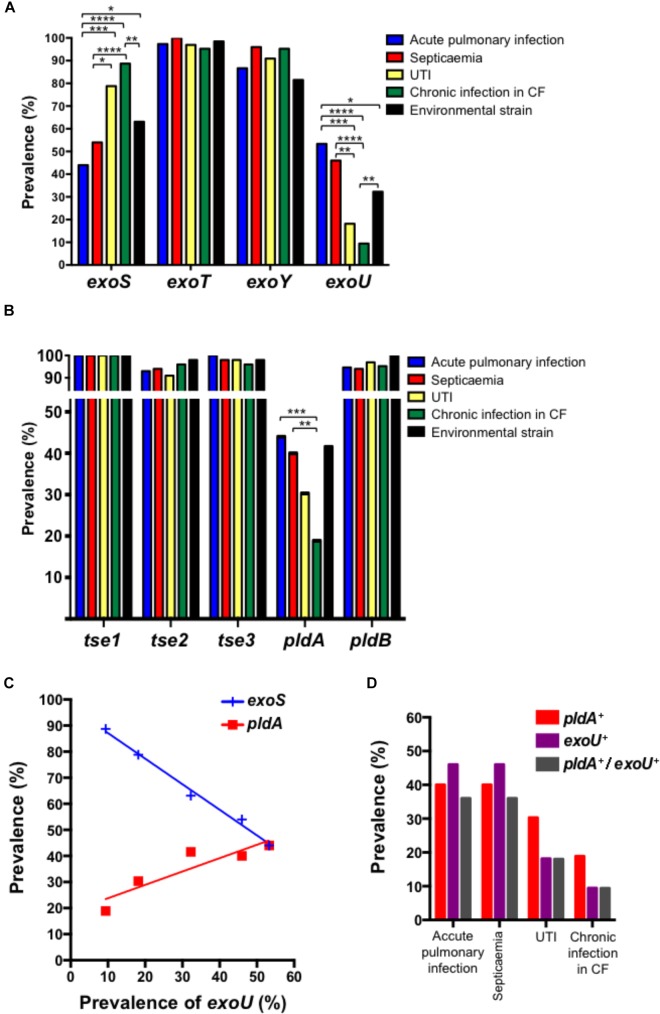
Prevalence of T3SS effectors **(A)** and T6SS effectors **(B)** in *Pseudomonas aeruginosa* isolates recovered from acute pulmonary infections, septicaemia, UTIs, chronic infections in CF patients and environment. Correlation between the prevalence of *exoS*, *exoU*, and *pldA* is indicated in **(C)**. Data were compared two-by-two using *χ*^2^ test (^∗^*p* < 0.05; ^∗∗^*p* < 0.01; ^∗∗∗^*p* < 0.001; and ^∗∗∗∗^*p* < 0.0001). **(D)** Prevalence of *pldA*, *exoU* and *pldA*^+^ *exoU* in *Pseudomonas aeruginosa* isolates recovered from accute pulmonary infections, septicaemia, UTIs and chronic infections in CF patients.

Among the 339 *P. aeruginosa* isolates the global prevalences of genes encoding T3SS-secreted exotoxins were 65.2% (221/339) for *exoS*, 98.5% (334/339) for *exoT*, 32.4% (110/339) for *exoU*, and 90.0% (305/339) for *exoY*. Regardless of the biological sample (urine, pulmonary sample, blood, and environmental samples) from which the strain was cultured, the prevalences of *exoT* and *exoY* were high, ranging from 95.2 to 100% for *exoT* and 86.2 to 95.9% for *exoY* (Figure 1A). As previously observed, we identified an inverse correlation between the presence of *exoS* and *exoU* in *P. aeruginosa* isolates (Figure 1C; Feltman et al., 2001). Of note, the prevalence of *exoU* was higher in invasive acute infections (53.3% in acute pulmonary, infection from non-CF patients and 46.0% in septicaemia) compared to environmental strain (32.3%), UTIs (18.2%) and chronic infection in CF patients (5.4%).

Regarding T6SS effectors, H1-T6SS effectors were highly prevalent in all *P. aeruginosa* isolates. Indeed, we observed prevalences of 100%, 91–98%, and 96–100% for *tse1*, *tse2*, and *tse3*, respectively (Figure 1B). This prevalence did not depend on the sample from which the strain was isolated. The same observation was made for *pldB* (H3-TSS6 effector) with a global prevalence of 96.8% (328/339), ranging from 94% in *P. aeruginosa* isolates responsible for acute pulmonary infection in non-CF patient to 100% in environmental strains. The prevalence of *pldA* was higher in strains responsible for acute invasive infection (acute pulmonary infection and septicaemia) as compared to UTI, environmental strain and strains isolated from chronic infection in CF patients (Figure 1B). Accordingly, we observed a correlation between *pldA* and *exoU* and consequently an inverse correlation with *exoS* (Figure 1C). Despite *pldA*^+^ and *exoU*^+^ correlation was found, the mutual exclusivity which was observed between *exoS*^+^ and *exoU*^+^ do not exist between *pldA*^+^ and *exoU*^+^ (Figure 1D).

### Increased Prevalence of *pldA* and *exoU* With Antimicrobial Resistance in Clinical Isolates

**FIGURE 2:**
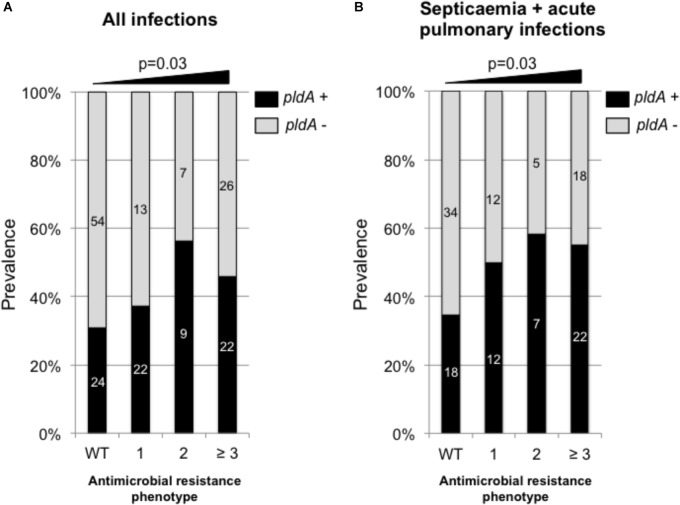
Prevalence of *pldA* in *P. aeruginosa* responsible for all type of infections (acute and chronic) **(A)** and acute infections **(B)** depending on the antimicrobial resistance phenotype. WT, wild-type phenotype; 1, resistance to one antimicrobial family, 2, resistance to two antimicrobial families; ≥3, resistance to three and more than three antimicrobial families. The antimicrobial families considered in the analysis were: penicillins (ticarcillin and piperacillin), broad-spectrum cephalosporins (ceftazidime and cefepime), carbapenems (imipenem and meropenem), fluroroquinolones (ciprofloxacine) and aminoglycosides (amikacin, tobramycin, and gentamicin). Raw numbers are indicated in side the bars. Statistical analysis was performed using *χ*^2^ test for trend.

Using our collection of 185 *P. aeruginosa* isolates responsible for infections we found that the *pldA* negative isolates were significantly more susceptible to antimicrobial than *pldA* positive isolates with a percentage of wild-type phenotype of 49.3% for *pldA* negative strain versus 30.5% for *pldA* positive isolates (χ^2^ test, *p* = 0.03). Of note, the tendency of being more resistant for *pldA*^+^ positive isolates was significant (χ^2^ test, *p* = 0.03) for all type of infections including acute infections (UTIs, pulmonary infections and septicaemia) and chronic infections in CF patients (Figure 2A). However, this tendency is exacerbated in acute infection only (Figure 2B). Since *exoU*^+^ and *pldA*^+^ were found to be correlated (Figure 1C), the same type of correlation between *exoU*^+^ and antimicrobial resistance was observed. Indeed, the percentage of *exoU*^+^ increased from 24.6% in isolates with wild-type phenotype to 43.6% on isolates harboring resistance to at least 3 antimicrobial families (data not shown).

To validate this hypothesis of increased prevalence of *pldA* positive *P. aeruginosa* isolates with the antimicrobial resistance, we tested our collection of carbapenemase-producing strains. In this collection of 33 extremely drug resistant isolates, the prevalence of *pldA* positive strain was maximum as compared to the other phenotypes with a global prevalence at 56.8%. Again, the prevalence of *exoU*^+^ strains followed the same overall tendency with a global prevalence of 41.8% in this extremely drug resistant isolates, close to the 43.6% observed previously with isolates harboring resistance to at least 3 antimicrobial families.

### IMP-Type Carbapenemase Production Is Associated With *pldA* Positivity

Of note, in our collection of 33 extremely drug resistant *P. aeruginosa*, all isolates that produce a carbapenemase of IMP-type (4 IMP-1, 1 IMP-2, and 4 IMP-13) were *pldA* positive whereas the prevalence of *pldA* was of 41.6% with other carbapenemase types (VIM, NDM, GES, SPM, and AIM). Among these IMP-producers, all IMP-13-producing isolates were of the same ST, the worldwide disseminated ST 621 (Santella et al., 2010). The other isolates were of ST 244-like and 304. More systematically, we looked for *pldA* gene in complete genomes of IMP-producing *P. aeruginosa* available in NCBI. Among the 107 complete genomes of *P. aeruginosa*, we identified four additional isolates producing IMP-1 (one ST-2613 and one ST-357) and two IMP-34 (both ST-2613). This analysis confirmed the systematic association between *pldA* presence and IMP-type carbapenemase production.

As opposed to *pldA*, *exoU* positivity was not associated with a specific carbapenemase type. Indeed, the prevalence of *exoU*^+^ was not significantly different in IMP-producers (30%) and in other carbapenemase types producers (29.4%).

## Discussion

Regarding of *P. aeruginosa* virulence factors during infection, the implication of T3SS-effectors has been deeply demonstrated (Feltman et al., 2001; Garey et al., 2008; Hauser, 2009). Our results regarding the prevalence of Exo toxins encoding genes are in accordance with previous studies that found prevalence of 58–78% for *exoS*, 92% for *exoT*, 89% for *exoY* and 28–48% for *exoY* in *P. aeruginosa* clinical isolates (Hauser, 2009). An inverse correlation between the presence of *exoS* and *exoU* in *P. aeruginosa* isolates has been previously demonstrated (Feltman et al., 2001). Our results confirm this inverse correlation (Figure 1). Of note *exoU*^+^ isolates are also considered to be more virulent (Schulert et al., 2003) and more often associated with severe acute infections, such as septicaemia (Wareham and Curtis, 2007) and severe acute pulmonary infections (Sullivan et al., 2014). Our results confirm the higher prevalence of *exoU*^+^ *P. aeruginosa* isolates in acute pneumonia and septicaemia. In addition, we also identified a correlation between *pldA* and *exoU* expression (Figure 1). Together, our results confim those of a very recent study which reports that the presence of *pldA* and *exoU* in mucoid *P. aeruginosa* is associated with high risk of exacerbations in non-CF patients (Luo et al., 2018). Since it has been demonstrated that PldA is a *trans*-kingdom T6SS effector that allows *P. aeruginosa* to interact with epithelial cells (Jiang et al., 2014; Hachani et al., 2016), we might hypothesize that the worst outcome observed with *exoU*^+^ isolates might also depend on the expression of *pldA*, at least partially. Interestingly, it has been recently described that the expression of T6SS encoding genes is up-regulated in presence of respiratory mucus (Cattoir et al., 2013), suggesting a potential role of T6SS during the infectious process. Unfortunately, the studied *P. aeruginosa* isolate was *pldA*^-^, and only the H1-T6SS genes expression was reported. Since we showed that *pldA* is more prevalent in isolates responsible for acute pulmonary infection, septicaemia and in a lesser extend UTIs, further studies are necessary to unravel whether H2-T6SS genes including *pldA* are also overexpressed in presence of relevant biological fluids (respiratory mucus, blood, and urine). In addition, one of the limitations of our study is the lack of information regarding the clinical outcomes of patients. Indeed, these data might be useful to identify any relevant clinical impact of PldA during infectious process. However, the suggested role of *pldA* in pulmonary infection is in agreement with the results of Wilderman et al. (2001). Indeed, they first identified that not all strains of *P. aeruginosa* carry *pldA* thus suppose to be part of a ∼7 kb mobile genetic element. In this study, *pldA* was found to be most prevalent in non-CF patients than in CF patients. Unfortunately, no data were presented regarding the type of infections in non-CF patients. Then, using competition studies between PldA knock-out mutant and its PAO1 parental strain, they demonstrated the contribution of PldA during chronic pulmonary infection in a rat model.

Unlike *pldA* results, no difference in the prevalence of *pldB*, another *trans*-kingdom T6SS that was demonstrated to interact with the host cells (Russell et al., 2013). Indeed, we found that *pldB* is barely present in all *P. aeruginosa* isolates. This finding is not in favor of a specific role of this phospholipase during certain types of infection.

Finally, as it was previously observed for *exoU* (Garey et al., 2008; Mitov et al., 2010), we found that *pldA*^+^ isolates were significantly more resistant than *pldA*^-^ isolates. We demonstated that *pldA* prevalence was maximum (∼60%) in extremely resistant *P. aeruginosa* isolates that produce a carbapenemase. Interestingly, among carbapenemase-producers, all IMP-type-producing strains were *pldA*^+^. Further studies are now needed to explore this close association between *pldA* and *bla*_IMP_ genes, particularly in the context of the worldwide dissemination high-risk clone such as IMP-13-producing *P. aeruginosa* of ST621 that are responsible for outbreaks of UTIs (Pagani et al., 2005; Santella et al., 2010).

To our knowledge, this is the first study that evaluated the prevalence of genes encoding a large subset of T6SS effectors, including the more recently described *pldA* and *pldB* genes, in clinical isolates of *P. aeruginosa*. We found that *pldA*, but not *pldB*, prevalence is increased in clinical isolates responsible for severe acute infection and particularly in multi-drug resistant isolates. In addition the hypervirulent *exoU*^+^ isolates are more prone to be *pldA*^+^, thus suggesting PldA might also play a role in this increased virulence. Finally, we showed that extremely drug resistant *P. aeruginosa* isolates that produce an IMP-type carbapenemase were all *pldA*^+^, an unexpected relationship that needs to be further explored.

## Ethics Statement

Written informed consent was not required as the samples were not recovered specifically for the study and no information regarding the patients were included in this study.

## Author Contributions

LD and TB had full access to all of the data in the study, and took responsibility for the integrity of the data and the accuracy of the data analysis. LD contributed to the study of concept and design. LD, TB, and Y-MB acquired, analyzed, or interpreted the data. LD, TB, and TN drafted the manuscript. LD, PP, AF, and TN contributed to critical revision of the manuscript for important intellectual content.

## Conflict of Interest Statement

The authors declare that the research was conducted in the absence of any commercial or financial relationships that could be construed as a potential conflict of interest.

## Footnotes

1 http://www.eucast.org/clinical_breakpoints/

2 www.ebi.ac.uk/ena
